# Risk of Neurodegenerative Diseases in Elderly Koreans with an Initial Diagnosis of Type 2 Diabetes: A Nationwide Retrospective Cohort Study

**DOI:** 10.1155/2023/7887792

**Published:** 2023-11-08

**Authors:** Hee-Cheol Kim, Ho-Jun Lee, Yang-Tae Kim, Byeong-Churl Jang

**Affiliations:** ^1^Department of Psychiatry, Keimyung University School of Medicine, Daegu 42601, Republic of Korea; ^2^Brain Research Institute, Keimyung University School of Medicine, Daegu 42601, Republic of Korea; ^3^Department of Molecular Medicine, Keimyung University School of Medicine, Daegu 42601, Republic of Korea

## Abstract

Type 2 diabetes (T2D) and neurodegenerative diseases (NDs) are common among elderly individuals. Growing evidence has indicated a strong link between T2D and NDs, such as Alzheimer's disease. However, previous studies have limitations in exploring the epidemiological relationship among these diseases as a group of NDs rather than as a specific type of ND. We aimed to investigate the risk of NDs in elderly Koreans who were first diagnosed with T2D and determine the association between T2D and NDs. We conducted a retrospective longitudinal cohort study of patients with who were initially diagnosed with T2D using the Korean National Health Information Database. The study participants were categorized into a T2D group (*n* = 155,459) and a control group (*n* = 155,459), aged 60–84 years, that were matched for age, sex, and comorbidities. We followed the participants for 10 years to investigate the incidence of NDs. The Cox proportional hazards regression model was used to estimate the hazard ratios (HRs) and 95% confidence intervals (CIs) for NDs. The numbers of patients diagnosed with ND at the end of follow-up were as follows: 51,096/155,459 (32.9%) in the T2D group and 44,673/155,459 (28.7%) in the control group (*χ*^2^ = 622.53, *p* < 0.001). The incidences of NDs in the T2D and control groups were 44.68 (95% CI: 44.29, 45.07) and 36.89 (95% CI: 36.55, 37.24) cases per 1,000 person-years at risk, respectively. The overall incidence of NDs was higher in the T2D group than that in the control group (HR: 1.23, 95% CI: 1.22, 1.25, *p* < 0.001). This study revealed a higher incidence of NDs in elderly Koreans who were initially diagnosed with T2D. This suggests that T2D is a risk factor for NDs in elderly Koreans.

## 1. Introduction

Neurodegenerative diseases (NDs) represent a large group of neurological diseases, including Alzheimer's disease (AD), frontotemporal dementia, Parkinson's disease (PD), and Huntington's disease. Although this group of diseases has different clinical and pathological phenotypes, they share important common pathological features characterized by age-dependent and progressive degeneration of neurons resulting from the accumulation of misfolded proteins [1]. Type 2 diabetes (T2D) has a heterogeneous pathophysiology, with complex interactions between age-related physical conditions and a wide range of risk factors [2, 3]. T2D and NDs are age-associated degenerative diseases that frequently occur in the elderly population, and various studies have been conducted on their pathophysiology. Aging is characterized by a gradual breakdown of the physiologically intact state, which causes functional problems and increases the likelihood of death. Among the many factors associated with human aging, mitochondrial dysfunction has emerged as a key feature of aging pathology and is associated with numerous age-related diseases [4–6]. Aging has also been linked to impaired protein expression or proteostasis [7]. Loss of proteostatic control can lead to the accumulation and aggregation of misfolded proteins, and these misfolded protein aggregates play an important role in cell dysfunction and tissue damage, leading to some age-related diseases, including T2D and NDs [8, 9]. Many studies have reported that these age-related diseases share a common pathophysiology; therefore, they are likely to be epidemiologically related.

Several epidemiological studies have demonstrated an association between T2D and ND, such as AD or PD have been reported [10, 11]. AD is the most common type of ND, and the relationship between T2D and AD was first reported in the Rotterdam Study, a prospective population-based cohort study. Of 6,370 elderly participants with an average follow-up of 2.1 years, 126 developed dementia, 89 of whom were diagnosed with AD [12]. A recent epidemiological study in Korea estimated the incidence of dementia in patients with T2D older than 40 years of age, with an average follow-up of 5.1 years; the results showed that the incidence rates of all types of dementia, AD, and vascular dementia in participants with diabetes were 9.5, 6.8, and 1.3 per 1,000 person-years, respectively [13].

Most previous studies have reported an association between T2D and certain types of ND. However, these studies have limitations in exploring the epidemiological relationship among these diseases as a group of NDs rather than as a specific type of ND, which is assumed to have a common pathophysiology. Therefore, we aimed to explore the epidemiological relationship between T2D and NDs. For this purpose, we conducted a retrospective longitudinal cohort study to estimate the incidence of NDs as a group of diseases in elderly patients with T2D and a matched control group, using the National Health Insurance Service (NHIS) database (DB) for the entire Korean population.

## 2. Materials and Methods

### 2.1. Ethics Statements

We obtained permission from the National Health Insurance Corporation Review Committee to use the National Health Information Data (NHIS-2021-1-253). This study was conducted in accordance with the ethical principles outlined in the Declaration of Helsinki. All research procedures and ethical aspects were approved by the Institutional Review Board (IRB) of Keimyung University Dongsan Hospital (IRB number: DSMC 2019-08-067-004).

### 2.2. Data Source

The data collected by the NHIS DB include detailed treatment practices and prescriptions, as well as the medical information of all citizens who signed up for medical insurance in Korea. NHIS claims for inpatient and outpatient visits, procedures, and prescriptions were coded using the International Classification of Diseases, 10^th^ Revision (ICD-10) and the Korean Drug and Anatomical Therapeutic Chemical Codes [14]. The NHIS routinely audits claims, and the data are considered reliable and used in numerous peer-reviewed publications [15, 16].

### 2.3. Study Design and Population

This study used the NHIS-Customized Data (NHIS-2021-1-253) for 14 years, from 2002 to 2015, including the NHIS health insurance and long-term care insurance data for the entire population of Korea. We selected data (study candidate group) from patients aged 60–84 years as of 2002 among those who had no diagnostic codes for T2D or any NDs in 2002. Diagnoses in the NHIS DB are based on the Korean Classification of Disease, 6^th^ version (KCD-6), which is a revision of the ICD-10. Diagnoses obtained from the medical insurance claims data may not be as accurate as those obtained from medical charts. Therefore, to increase the diagnostic accuracy, T2D was defined as KCD-6 codes with E11 assigned more than twice per patient. NDs were defined as patients with more than one medical claim with a diagnostic code of AD, PD, or circumscribed brain atrophy (CBA) in the main or auxiliary diagnosis (including the first through third). The KCD-6 codes for AD include G300, G301, G308, G309, F000, F001, F002, or F009. The KCD-6 codes for PD include G20 and F023. The KCD-6 codes for CBA include G310, G3101, G3102, G3103, G3104, G3108, and F020.

**Figure 1:**
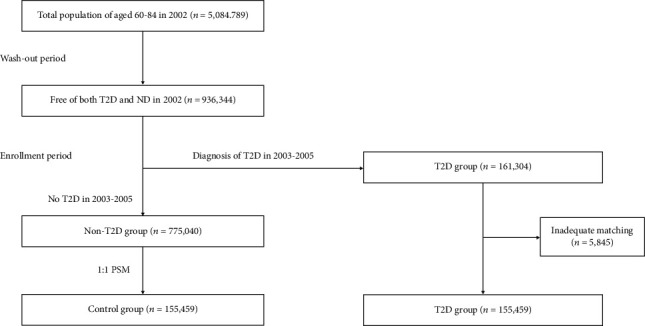
The flow process of the sampling method. T2D: type 2 diabetes; ND: neurodegenerative disease; PSM: propensity score matching.

The flowchart of the sampling method is illustrated in Figure 1. Among patients who had never been diagnosed with any NDs during the study enrollment period (2003–2005), those who were diagnosed with T2D for the first time were selected from the study candidate group as the T2D group (*n* = 161,304). Patients without a diagnosis of T2D or NDs during the study enrollment period were selected from the study candidate group as the non-T2D group (*n* = 775,040). Among the non-T2D group, the patients with similar characteristics to the T2D group, including age, sex, and comorbid diseases, were selected as the control group. We used a 1 : 1 propensity score matching (PSM) method to match patients in the non-T2D group with those in the T2D group. A total of 155,459 pairs (96.4% of patients in the T2D group and 20.1% of patients in the non-T2D group) were matched in both groups. Therefore, the total numbers of participants were 310,918 with 155,459 in each group. On the basis of the data extracted through this process, these patients were followed up for 10 years, from January 1, 2006, to December 31, 2015, and the incidence of NDs was investigated.

In the process of data extraction, propensity scores were calculated on the basis of the participants' age, sex, and comorbidities, as assessed using the Charlson Comorbidity Index (CCI) total score. The CCI is a scale designed to adjust for the severity of comorbidities [17]. It was originally developed as an index to adjust for the patient's risk of death in experimental studies, but it is also widely used as a method to adjust for the severity of comorbidities.

### 2.4. Data Management and Statistical Analysis

We present data for continuous variables as mean ± standard deviation and data for categorical variables as number with percentage. The comparison of continuous variables between the T2D and control groups was performed using Student's *t*-test. The chi-square test was used to determine the difference in the frequency of NDs between the T2D and control groups. The Kaplan–Meier survival curve analysis was used to estimate the cumulative incidence of NDs during the 10-year follow-up period, and the log-rank test was used to compare survival curves between the T2D and control groups. The proportional hazards (PH) assumption underlying both the log-rank test and the Cox regression model was examined graphically, and the results showed no violations. Multivariable Cox proportional hazards regression analysis was used to estimate the adjusted hazard ratios (HRs) and 95% confidence intervals (CIs) for the incidence of NDs after adjusting for confounding variables, including age, sex, comorbidities (CCI total score), and the annual average number of visits to medical institutions. Stata/MP 16 (StataCorp, LLC, College Station, TX, USA) was used to perform data management and statistical analyses. The level of statistical significance was set at *p* < 0.05.

## 3. Results and Discussion

### 3.1. Sample Characteristics

Before PSM, there were statistically significant differences in sex, age, and CCI total score between the T2D and control groups. However, after PSM, the difference between the two groups was not significant for sex. The differences in age and CCI total score between the two groups were minimal but still statistically significant (Table 1). Regarding the CCI total score, there was a statistically significant difference between the control and T2D groups, but the actual score difference in the clinical aspect was very small owing to the very large number of study participant. Several studies conducted on stroke patients indicated that a CCI score of 0-1 was considered a low comorbidity, whereas a score of 2 or higher was considered a high comorbidity [18–20].

### 3.2. Incidence of NDs

**Figure 2:**
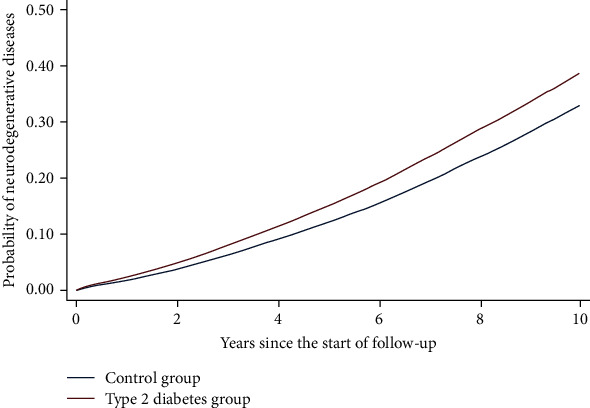
The Kaplan-Meier cumulative probability curves showing the incidence of neurodegenerative diseases in the control and type 2 diabetes groups.

As of December 31, 2015 (the end of the observation period), the numbers of patients with ND were 44,673/155,459 (28.7%) in the control group and 51,096/155,459 (32.9%) in the T2D group (*χ*^2^ = 622.53, *p* < 0.001). The incidences of NDs were 44.7 per 1,000 person-years (95% CI: 44.3, 45.1) in the T2D group and 36.9 per 1,000 person-years (95% CI: 36.6, 37.2) in the control group (Table 2).  Figure 2 shows the Kaplan–Meier survival curves for the cumulative incidence of NDs between the T2D and control groups. The log-rank test showed a significantly different pattern of survival curves between the two groups (*χ*^2^ = 1257.29, *p* < 0.001). To confirm the temporal trend of the difference in the incidence of NDs in the two groups during the 10-year follow-up period, Table 3 presents the results of comparing the incidence of NDs between the two groups by every 2-year interval.

The average age of the control group was 72.9 years, whereas that of the T2D group was 72.4 years, showing a statistically significant difference between the two groups. Therefore, we stratified age into four groups at 5-year intervals and calculated the incidence rate ratio of ND according to the age strata. The incidence of ND was significantly higher in the T2D group than in the control group in all four age groups (Table 4).

After adjusting for age, sex, comorbidities (CCI total score), and the annual average number of visits to medical institutions, the adjusted HR for NDs in the T2D group compared with that in the control group was 1.23 (95% CI: 1.22, 1.25, *p* < 0.001). In addition, we separately evaluated the adjusted HR for a specific type of ND in the T2D group compared with that in the control group. The adjusted HRs were 1.29 for AD (95% CI: 1.27, 1.31; *p* < 0.001), 1.44 for PD (95% CI: 1.39, 1.50, *p* < 0.001), and 1.09 for CBA (95% CI: 1.07, 1.12, *p* < 0.001).

From the perspective of shared pathophysiology, we considered several types of NDs as a single ND group to investigate the epidemiological association between T2D and NDs. The incidence of NDs was significantly higher in the T2D group than in the control group. This study showed a 14.6% increase in the number of patients with ND in the T2D group compared with that in the control group over a 10-year follow-up period. This study also showed that T2D was positively associated with NDs, supporting our assumption that T2D and NDs share a common pathophysiology.

It is difficult to directly compare the results of this study with those of previous studies, because no previous study has analyzed all NDs as a single ND group. We compared and reviewed the results of many previous studies on the association between T2D and specific ND, such as AD or PD. Previous studies have reported an association between T2D and AD, and various vascular factors (hypertension, diabetes, heart disease, and current smoking) are involved in the association between these two diseases, with diabetes and smoking being the strongest risk factors. The risk of AD associated with diabetes was much higher than that previously reported (relative risk, 3.8) [21]. Cheng et al. [22], in a cohort study population recruited in Manhattan between 1992 and 1994 and followed from 1999 to 2001, demonstrated that T2D was strongly associated with late-onset AD, even after adjusting for sex and age. Their study also suggested that the association between T2D and late-onset AD was mediated, in part, by cerebrovascular pathology. A study by Li et al. [23] reported that T2D affects the progression to AD in an elderly Chinese population with mild cognitive impairment; however, no change was observed in an age-matched control group. These findings are supported by longitudinal studies conducted by Leibson et al. [24] and Huang et al. [25], in which patients with adult-onset diabetes developed AD more frequently than age-matched controls without T2D.

Many studies have also been published on the relationship between PD, the most common form of ND after AD, and T2D. A meta-analysis conducted in 2011 showed that diabetes acts as a risk factor for PD in a prospective study (risk ratio (RR): 1.37, 95% CI: 1.21, 1.55, *p* < 0.001) [26]. A subsequent meta-analysis included seven cohort studies involving more than 1.7 million individuals and concluded that the PD risk of approximately 38% in patients with diabetes was increased (RR: 1.38, 95% CI: 1.18, 1.62, *p* < 0.001) [27]. According to a recent systematic review and meta-analysis of observational studies, pooled effect estimates showed that T2D was associated with an increased risk of PD (odds ratio (OR): 1.21, 95% CI: 1.07, 1.36), and there was some evidence that T2D was associated with faster progression of motor symptoms (standardized mean difference (SMD): 0.55, 95% CI: 0.39, 0.72) and cognitive decline (SMD: -0.92, 95% CI: -1.50 to -0.34) [28]. In this study, the incidence of NDs was higher in T2D patients than in controls, and this result was confirmed for all NDs, including AD, PD, and CBA. The results of the present study and those of many previous epidemiological studies support the possibility that T2D and several NDs are related and share the same pathophysiology.

T2D and NDs are typical age-related diseases related to impaired proteostasis and aggregation of damaged proteins [29, 30]. Protein misfolding is a common consequence of proteostatic failure and a key contributor to many NDs and other disorders. Recently, it was reported that failure of proteostasis may contribute to cancer development and progression by disrupting the normal function of critical proteins involved in cell growth regulation, DNA repair, and other processes [31, 32]. If T2D, NDs, and cancer, which are common in old age, share some common pathophysiological mechanisms such as failure of proteostasis, these diseases are likely to be related to each other. In a previous study, we found a positive association between NDs and cancer, which was additively influenced by T2D [33].

Although proteostasis failure has been implicated in several age-related diseases, it remains poorly understood how it leads to pathological events in the intracellular network regulating proteostasis. Recent studies have reported a link between mitochondrial dysfunction and proteostasis failure [34, 35]. Mitochondrial dysfunction is also associated with several age-related diseases, including T2D, NDs, cardiovascular diseases, and cancer [4–6, 36]. T2D, NDs, and cancer are distinct medical conditions with different phenotypes. However, common underlying factors may contribute to these conditions. It is also important to note that although there may be commonalities, each condition has its own unique set of causes and pathophysiological processes. In the future, large-scale epidemiological studies are needed to determine the relationship between these three diseases, and studies are also needed to identify the biological factors that mediate these diseases.

This study had some limitations. First, this study had a retrospective observational design based on medical claim data, and the diagnoses for diseases identified using KCD-6 codes in the claims database may have been inaccurate compared with diagnoses obtained from medical charts, imaging results, or laboratory findings. Second, we did not control for personal history such as diet, exercise, alcohol consumption, or smoking, which could influence the pathogenesis of the disease. Despite these limitations, this study has the strength of being a cohort study based on a large population with a 10-year follow-up period.

## 4. Conclusions

This study revealed a higher risk of developing NDs in elderly Koreans with T2D than in those without T2D. These results suggest that T2D is a risk factor for NDs in elderly Koreans and support our assumption that age-related diseases such as T2D and NDs will have a common pathophysiology.

## Figures and Tables

**Table 1:** Characteristics of study subjects before and after propensity score matching (PSM).

Variables	Before PSM			After PSM		
	Control group	T2D group	*p* value	Control group	T2D group	*p* value
Sex			<0.01			0.46
Male (%)	385,783 (49.8)	77,716 (48.2)		76,091 (49.0)	75,885 (48.8)	
Female (%)	389,257 (50.2)	83,588 (51.8)		79,368 (51.0)	79,574 (51.2)	
Age	72.91 ± 5.60	72.35 ± 5.24	<0.01	72.31 ± 5.26	72.16 ± 5.20	<0.01
CCI total	0.23 ± 0.51	0.52 ± 0.85	<0.01	0.46 ± 0.77	0.48 ± 0.83	<0.01

T2D: type 2 diabetes; CCI: Charlson comorbidity index.

**Table 2:** Person-years incidence rates of ND over a 10-year follow-up period.

Group	Person-years	No. of ND	Rate^∗^	95% CI
Control	1,210,934.31	44,673	36.89	36.55~37.24
T2D	1,143,667.45	51,096	44.68	44.29~45.07
Total	2,354,601.76	95,769	40.67	40.42~40.93

^∗^Rate means incidence rate (per 1000 person-years). ND: neurodegenerative disease; CI: confidence interval; T2D: type 2 diabetes.

**Table 3:** Person-years incidence rates of ND at 2-year intervals during the 10-year follow-up period.

Group	Person-years	No. of ND	Rate^∗^	95% CI
Control				
0-2	303,152.33	5,841	19.27	18.78~19.77
2-4	276,270.47	7,848	28.41	27.79~29.04
4-6	244,807.13	8,924	36.45	35.70~37.22
6-8	210,789.92	10,781	51.15	50.19~52.12
>8	175,914.46	11,279	64.12	62.94~65.31

T2D				
0-2	300,778.76	7,528	25.03	24.47~25.60
2-4	267,524.36	9,547	35.69	34.98~36.41
4-6	230,141.37	10,357	45.00	44.14~45.88
6-8	191,354.26	12,048	62.96	61.85~64.10
>8	153,868.70	11,616	75.49	74.13~76.88
Total	2,354,601.76	95,769	40.67	40.42~40.93

^∗^Rate means incidence rate (per 1000 person-years). ND: neurodegenerative disease; CI: confidence interval; T2D: type 2 diabetes.

**Table 4:** Incidence rate of ND stratified by age group in control and T2D groups.

Age range	Control		T2D			
	No. of ND	Rate^∗^	No. of ND	Rate^∗^	IRR^†^	95% CI
All age	44,673	36.89	51,096	44.68	1.21	1.20~1.23
65~69	11,003	22.78	13,872	29.46	1.29	1.26~1.33
70~74	14,041	35.58	16,887	44.80	1.26	1.23~1.29
75~79	11,442	53.30	12,244	63.75	1.20	1.17~1.23
80 ≤	8,187	69.05	8,093	77.97	1.13	1.10~1.16

^∗^Rate means incidence rate (per 1000 person-years). ND: neurodegenerative disease; T2D: type 2 diabetes; IRR: incidence rate ratio; CI: confidence interval. ^†^*χ*^2^ = 53.62, *p* < 0.001.

## Data Availability

The datasets that support the findings of this study are not readily available because the data analyzed in this study are stored on the National Health Insurance Corporation server. The data can only be accessed and used remotely from a designated location within the National Health Insurance Corporation and cannot be retrieved externally.
